# Energetics of Underwater Swimming in Apnea

**DOI:** 10.1249/MSS.0000000000003731

**Published:** 2025-04-15

**Authors:** GIOVANNI VINETTI, ANNA TABONI, NAZZARENO FAGONI, ENRICO TAM, CARSTEN LUNDBY, GUIDO FERRETTI

**Affiliations:** 1 Department of Molecular and Translational Medicine, University of Brescia, Brescia, ITALY; 2 Institute of Mountain Emergency Medicine, Eurac Research, Bolzano, ITALY; 3 Department of Anaesthesiology, Pharmacology, Intensive Care, and Emergency Medicine, University of Geneva, Geneva, SWITZERLAND; 4 Department of Neuroscience, Biomedicine and Movement, University of Verona, Verona, ITALY; 5 Faculty of Health Sciences, Department of Sports Science and Clinical Biomechanics, University of Southern Denmark, Odense, DENMARK

**Keywords:** ALVEOLAR GAS, BREATH-HOLD DIVING, FREEDIVING, SWIMMING ECONOMY, AEROBIC METABOLISM, ANAEROBIC METABOLISM

## Abstract

**Purpose:**

Dynamic apnea with fins (DYN) involves swimming the longest distance relying solely on the body’s oxygen and anaerobic energy stores. The energy cost per unit distance (*C*) is therefore an important determinant of DYN performance, yet it has never been measured. This study aimed to assess the *C* of DYN and its aerobic (EO_2_), anaerobic lactic (ELa), and alactic (EPCr) energy contributions.

**Methods:**

In a 50-m swimming pool, 22 freedivers (3 females; 10 using bi-fins, 6 using monofin, 6 using both) performed a 50-m DYN, and 7 performed a 100-m DYN. Net *C* (above resting) was calculated from the O_2_ debt measured at emersion plus ELa (calculated from the blood lactate increase). In nine subjects (six of whom performed also the 100-m DYN), determination of hemoglobin mass and total lung capacity allowed the estimation of EO_2_ and, by subtraction, EPCr.

**Results:**

*C* was unchanged between the 100-m and the 50-m DYN (*P* = 0.81) and resulted higher with bi-fins than with the monofin (7.4 ± 2.2 vs 5.5 ± 1.6 J·kg^−1^·m^−1^, *P* = 0.02) due to a higher O_2_ debt and ELa. DYN personal best correlated better with the distance swum per unit of EO_2_ at 50 m (*R*^2^ = 0.70) than with *C* (*R*^2^ = 0.25). From 50 to 100 m, fractional EO_2_ decreased (58% ± 19% to 47% ± 13%, *P* = 0.02), ELa increased (10% ± 5% to 21% ± 5%, *P* < 0.001), and EPCr was unchanged (31% ± 20% to 32% ± 15%, *P* = 0.83).

**Conclusions:**

The *C* of DYN seems compatible with published values for surface swimming with fins at the same speed. At 100 m, ELa and EPCr were disproportionately high for the exercise intensity, possibly due to a diving response. Sparing EO_2_ is at least as important as *C* in determining DYN performance.

The energetics of surface swimming has been determined in several studies, and its characteristics are well established (1,2). Today, in addition to classic swimming specialties, disciplines are emerging that involve freediving under the surface of the water. However, the energetics of underwater swimming in apnea has not been determined so far. No data are available on its energy cost or on the systematic partitioning of the contribution of aerobic (EO_2_), anaerobic lactic (ELa), and anaerobic alactic (EPCr) energy systems. To date, the only known bioenergetic feature of underwater apneic swimming is the higher blood lactate concentration ([La]) compared with surface swimming (3–6), which increases with swimming speed (7) and decreases with breath-hold training status (8,9). The energetics of underwater fin swimming has only been characterized in eupneic conditions while wearing a self-contained underwater breathing apparatus (SCUBA) (10–14). The lack of studies on its apneic counterpart is surprising, as this practice has become very popular for recreational and competitive purposes. Horizontal underwater swimming in apnea is a growing sport discipline, known as dynamic apnea (DYN), whose competitive goal is to swim the greatest distance on a single breath (15).

The speed of a human in motion is equal to the ratio of the metabolic power to the energy cost per unit distance (*C*). Thus, the distance covered in a given time is equal to the ratio of the metabolic energy (*E*) to *C*. Concerning freediving, the maximum distance is directly proportional to the maximum exploitable stored *E* compatible with consciousness and inversely proportional to the *C* of underwater swimming (*d*_max_ = *E*_max_/*C*). Therefore, *C* is considered a key factor affecting maximum DYN distance (15) or dive depth (16). To minimize *C*, several pieces of equipment are used, including i) fins, with the monofin being preferred over bi-fins at the elite level due to its greater efficiency (17), and ii) a neck counterweight to maintain neutral buoyancy and counteract the leg-sinking torque (15).

Assessing *E* during apneic exercise is challenging due to the evident impossibility of using open-circuit respirometry techniques until the subject has resumed breathing. Previous studies estimated *E* during repetitive submaximal breath-hold dives, typical of sea harvesters, from the post-emersion excess O_2_ consumption (V̇O_2_) above resting baseline (18–21). In one of these studies, the increase in [La] was also accounted for (21). Of note, the cumulative excess V̇O_2_ during recovery was larger than that needed to replete body O_2_ stores, and the remainder has been attributed to the reconstitution of the phosphocreatine (PCr) depleted during the dive (22). In elite breath-hold divers during submaximal deep dives, Ferretti et al. (23) quantified EO_2_ from changes in measured lung plus estimated blood O_2_ stores and ELa from the changes in [La]. However, it was not possible to estimate EPCr as post-emersion excess V̇O_2_ was not measured.

The primary aim of this study was to determine the energy cost of dynamic apnea with bi-fins and the monofin by measuring the post-emersion excess V̇O_2_ and [La] accumulation. We hypothesized that *C* would be lower with the monofin compared with bi-fins (17) and, as full body submersion reduces wave drag (1), lower than in surface fin swimming at the same speed. A secondary aim was to assess the contribution of each of the three energy systems.

## METHODS

### Subjects

Twenty-two healthy competitive breath-hold divers (3 females, 41 ± 10 yr, 176 ± 7 cm, 76 ± 11 kg) with heterogeneous performance levels in DYN (personal best 122 ± 49 m with bi-fins and 150 ± 67 m with the monofin; overall range, 65–276 m) provided written informed consent to participate in the study, which was part of a larger project (24–26). The study was conducted in accordance with the Declaration of Helsinki and was approved by the local ethical committee. Subjects were instructed to avoid caffeine, alcohol, and heavy exercise in the previous 24 h and to eat a light meal 3–4 h before testing.

All participants performed a single-breath O_2_ dilution test (27) to assess total lung capacity (TLC), expressed at body temperature and pressure, saturated with water vapor (BTPS). This was performed in the supine position to mimic the increased thoracic blood volume characteristic of water immersion. A subgroup of nine subjects underwent measurement of hemoglobin mass (Hb_mass_) and blood volume (BV) with the optimized carbon monoxide rebreathing method (OpCO, Detalo, Denmark) (28,29), allowing the estimation of body oxygen stores.

### Experimental Trials

Trials were conducted in the side lane of a 50-m-long, 2.0-to 2.5-m-deep indoor Olympic swimming pool at sea level (Bella Italia Village, Lignano Sabbiadoro, Italy). Water temperature was 26.9°C ± 0.5°C, and ambient temperature and relative humidity were 27.3°C ± 1.0°C and 59% ± 6%.

Subjects were equipped with a neoprene wetsuit, swim cap, goggles, nose clip, bi-fins or monofin (according to individual preference), and a counterweight at the base of the neck. They immersed themselves in the pool up to chest level while standing on a 1.2-m-deep removable platform. They then performed their usual pre-dive routine, except that glossopharyngeal insufflation was not allowed to ensure that the starting lung volume matched the preliminarily measured TLC. After the final maximal inspiration, they wore the nose clip and started the DYN trial, swimming ~1 m below the water surface. Investigated DYN distances were 0 m (for baseline), 50 m, and, if the subject’s personal best was >120 m, 100 m. The 0-m trial was always performed first, whereas the 50- and 100-m trials were performed in random order. Subjects were instructed to maintain at every trial the same pre-dive hyperventilation, swimming technique, and speed as if they had to perform a maximal DYN in a competition.

At the end of the DYN, subjects reached the end wall of the pool, and the elapsed time (*t*) was recorded. Then, without resuming breathing, they raised the head above the water level, were quickly fitted with a face mask connected to a metabolic cart (Quark CPET; COSMED, Rome, Italy), and performed a deep exhalation, which was sampled at 50 Hz to assess the alveolar partial pressure of O_2_ and CO_2_ (P_A_O_2_ and P_A_CO_2_). The additional apneic time required for emersion and exhalation was also recorded (*t*_EE_). Simultaneously, both earlobes were exposed, dried, and warmed with a heated towel. One was immediately connected to a pulse oximeter (Nonin Medical, Plymouth, MN) to assess the nadir in peripheral O_2_ saturation (SpO_2_); the other was used to obtain serial 10-μL capillary blood samples at minutes 1, 3, 5, and 7 of recovery to assess peak [La] by an electroenzymatic method (Lactate Pro, Arkray, Japan). Post-emersion V̇O_2_ was continuously measured breath-by-breath with the same metabolic cart for 6 min, while the subjects recovered immersed, leaning against the side wall of the pool. This same procedure was followed also for the 0-m DYN, except that the subject never actually submerged; thus, the pre-dive routine was immediately followed by the aforementioned measurements, which were considered the pre-DYN baseline values.

### Total Energy Cost

**FIGURE 1:**
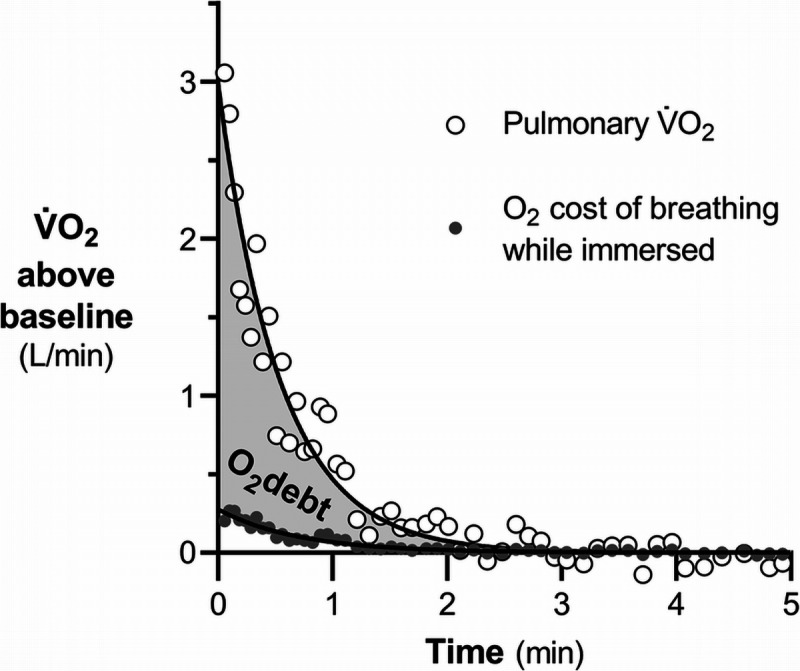
Post-emersion time course of whole-body O_2_ consumption (white dots) and the estimated O_2_ cost of breathing during head-out water immersion (gray dots) in a representative subject. For clarity, their respective asymptotic baselines have been subtracted so that the volume of oxygen used to restore the O_2_ debt contracted during the trials can be visualized as the gray area.

All metabolic energies are expressed in liters of oxygen at standard pressure and temperature, dry (STPD) unless otherwise stated. Post-apnea V̇O_2_ and expired ventilation were modeled with a mono-exponential decay function, excluding 1–2 initial outlier breaths (30). This procedure was not intended to accurately describe the V̇O_2_ kinetics, but rather to provide a robust estimate of the cumulative excess volume of O_2_ consumed during recovery as the area under the modeled V̇O_2_ curve above the asymptotic baseline. The post-emersion excess V̇O_2_ was attributed to i) the payment of the O_2_ debt contracted during the total breath-hold time (O_2_debt), and ii) the additional O_2_ consumed by the respiratory muscles above resting levels during recovery, due to the high ventilation rates with the thorax still submerged. The latter was estimated to be 3.5 mL O_2_ per liter of expired ventilation during head-out water immersion (31). O_2_debt was then calculated as the time integral of the modeled V̇O_2_ above baseline minus the one analogously calculated for the O_2_ cost of expired ventilation (Fig. 1).

ELa was calculated by multiplying the increase in [La] accumulation above the 0-m value by its energetic equivalent (3 mL O_2_ kg^−1^·mM^−1^) (32). O_2_debt includes the energy paid for the restoration of both the phosphocreatine pool (EPCr) and the oxygen stores (EO_2_), so the total energy expenditure (*E*) was calculated as:


$$E=EO_{2}+\text{EPCr}+\mathrm{ELa}=O_{2}\text{debt}+\mathrm{ELa}$$ 1

To isolate the metabolic power specific to DYN (*Ė*), the energy expended during the additional apneic time required for emersion and exhalation (*t*_EE_, 5 ± 2 s) was subtracted from *E*, assuming that these activities were mostly passive and thus required only resting metabolism (*Ė*_rest_):


$$\dot{E}=\frac{E-{\dot{E}}_{\text{rest}}\cdot t_{\mathrm{EE}}}{t}$$ 2

*Ė*_rest_ was calculated as the average V̇O_2_ from the minutes 3 to 6 after the 0-m DYN minus the O_2_ cost of breathing while immersed (31). This adjustment was made because resting V̇O_2_ was measured during water immersion, whereas there is no breathing during DYN. Finally, the net *C* of DYN was calculated as:


$$C=\frac{\dot{E}-{\dot{E}}_{\text{rest}}}{v}$$ 3

where *v* is the swimming speed. Due to the inherent absence of steady-state gas exchange data during DYN and recovery, an equivalent of 20.9 kJ (L O_2_)^−1^ (corresponding to a metabolic respiratory quotient of 0.96) was used as in previous swimming studies (1,17).

#### Aerobic and anaerobic alactic energy partitioning

Lung, muscular, and blood oxygen stores were considered (33). The decrease in lung oxygen stores (Δ*L*) was calculated according to (34):


$$\Delta L=\frac{\mathrm{TLC}-V_{D}}{863\;\mathrm{mm}\;\mathrm{Hg}\;}\cdot \left(P_{A}O_{2\mathrm{pre}}-P_{A}O_{2\text{post}}\;\frac{P_{A}N_{2\mathrm{pre}}}{P_{A}N_{2\text{post}}}\right)\;$$ 4

where 863 mm Hg is the combined conversion factor from BTPS to STPD and from partial pressure to gas fraction, *V*_D_ is the predicted anatomical dead space (35), the suffix “pre” refers to the 0-s DYN, and the suffix “post” to the 50- and 100-m DYN. Assuming conservation of the alveolar N_2_ mass, the P_A_N_2_ ratio yields the fractional change in alveolar volume after DYN.

The decrease in muscle oxygen stores (Δ*m*) was calculated assuming a myoglobin concentration of 5 g·kg^−1^ muscle (36), a muscle mass equal to 40% body weight (37), an O_2_-carrying capacity of 1.34 mL g^−1^, as for hemoglobin and a saturation decreasing linearly with DYN distance, from 90% at rest to 0% at the subject’s personal best.

The decrease in the arterialized blood oxygen stores (Δ*a*) was calculated assuming that arterialized blood represents 25% of BV (38):


$$\Delta a=0.25\cdot \left(1.34\cdot {\mathrm{Hb}}_{\text{mass}}\cdot \mathrm{\Delta Sp}O_{2}+\mathrm{BV}\cdot 0.03\cdot \Delta P_{A}O_{2}\right)$$ 5

where Hb_mass_ is expressed in grams and BV in liters and 0.03 is the O_2_ solubility in blood in mL L^−1^ mm Hg^−1^ (39).

The decrease in the venous oxygen stores (Δv̄) could not be measured. However, we estimated it via two different computational approaches. In the first (C1), we estimated Δv̄ and then derived EPCr by subtraction from O_2_debt. In the second (C2), we estimated EPCr and then derived Δv̄ by subtraction from O_2_debt. Regarding C1, we hypothesized that the increase in arteriovenous O_2_ difference from rest to end DYN, Δ(Ca − Cv̄) would follow the same kinetics as that invasively measured during a moderate exercise transition (40):


$$\Delta \left(\mathrm{Ca}-C\bar{v}\right)=k\cdot 49\;\frac{\mathrm{mL}}{L}\cdot \left(1-e^{-\frac{t-\delta }{\tau }}\right)$$ 6

where 49 mL L^−1^ is the average steady state Δ(Ca − Cv̄) in the exercise transition reported by Grassi et al. (40), *k* is a correction factor to account for different steady-state V̇O_2_ above baseline (ΔV̇O_2_), *δ* is the time delay, and *τ* is the time constant. Because steady-state Δ(Ca − Cv̄) is roughly proportional to the steady-state ΔV̇O_2_ in the investigated V̇O_2_ ranges, *k* is the ratio between our individual theoretical ΔV̇O_2_ and the average ΔV̇O_2_ of Grassi et al. (40) [*k* = (Ė − *Ė*_rest_ − ELa/*t*)/(1.72 mL min^−1^)], where ELa/*t* is the anaerobic lactic metabolic power of the trial. From the average data of Table 1 of Grassi et al. (40), we obtained *δ* = 14.5 s and *τ* = 12.9 s. Because their *δ* was measured in the femoral vein, we increased *δ* by 10 s (24.5 s) to account for the additional delay between the fall in femoral venous O_2_ concentration and the fall in the average venous O_2_ concentration of the whole venous volume (ΔCv̄O_2_). In so doing, the calculated ΔCv̄O_2_ can be applied to the whole venous volume (75% of BV) to obtain Δv̄:

**TABLE 1:** Measured parameters between fin types during the 50-m dynamic apnea.

	Bi-Fins (*n* = 16)	Monofin (*n* = 12)
Time (s)	48.0 ± 6.8	43.5 ± 10.9
Speed (m·s^−1^)	1.06 ± 0.17	1.22 ± 0.30
P_A_O_2_ (mm Hg)		
0 m	123 ± 6	127 ± 5
50 m	52 ± 10	56 ± 10
P_A_CO_2_ (mm Hg)		
0 m	29 ± 5	27 ± 4
50 m	49 ± 6	48 ± 4
SpO_2_		
0 m	100% ± 0%	100% ± 0%
50 m	84% ± 9%	85% ± 8%
[La] (mM)		
0 m	1.1 ± 0.1	1.0 ± 0.1
50 m	2.1 ± 0.9*	1.4 ± 0.4
ELa (L)	0.22 ± 0.19*	0.08 ± 0.08
O_2_debt (L)	1.47 ± 0.34*	1.17 ± 0.31
*E* (L)	1.69 ± 0.45**	1.25 ± 0.37
*Ė* (L min^−1^)		
0 m	0.37 ± 0.06	0.34 ± 0.06
50 m	2.11 ± 0.64	1.75 ± 0.53
*Ė* (mL·kg^−1^·min^−1^)		
0 m	4.8 ± 0.8	4.6 ± 1.0
50 m	28 ± 9	24 ± 8
*C* (kJ·m^−1^)	0.57 ± 0.18*	0.41 ± 0.14
*C* (J·kg^−1^·m^−1^)	7.4 ± 2.2*	5.5 ± 1.6
Δ*L* (L)	0.65 ± 0.16	0.65 ± 0.14

Ten subjects used bi-fins, six used the monofin, and six used both.

*P < 0.05 versus 50 m.

**P < 0.01 versus 50 m.


$$\Delta \bar{\mathrm{v̄}}\left(C1\right)=0.75\cdot \mathrm{BV}\cdot \mathrm{\Delta C}\bar{\mathrm{v̄}}O_{2}=0.75\cdot \mathrm{BV}\cdot \left[\mathrm{\Delta Ca}O_{2}+\Delta \left(\mathrm{Ca}-C\bar{\mathrm{v̄}}\right)\right]$$ 7

where ΔCaO_2_ is the measured decrease in arterial O_2_ concentration [Δ*a*/(0.25 BV)]. Thus, it is possible to calculate all the remaining parameters under C1:


$$EO_{2}\left(C1\right)=\Delta L+\Delta m+\Delta a+\Delta \bar{\mathrm{v̄}}\left(C1\right)$$ 8

$$\text{EPCr}\left(C1\right)=O_{2}\text{debt}-EO_{2}\left(C1\right)$$ 9

Regarding C2, we hypothesized that PCr kinetics would be the same as for a moderate exercise transition:


$$\text{EPCr}\left(C2\right)=\tau \cdot \left(\dot{E}-{\dot{E}}_{\text{rest}}\right)\cdot \left(1-e^{-\frac{t}{\tau }}\right)$$ 10

where *τ* was assumed equal to 23.3 s (41). On this basis, we estimated EO_2_ and Δv̄, respectively, as:


$$EO_{2}\left(C2\right)=O_{2}\text{debt}-\text{EPCr}\left(C2\right)$$ 11

$$\Delta \bar{\mathrm{v̄}}\left(C2\right)=O_{2}\text{debt}-\text{EPCr}\left(C2\right)-\Delta L-\Delta m-\Delta a$$ 12

Finally, the resulting mixed venous O_2_ concentrations resulting from the two pathways (Cv̄O_2_(C1) and Cv̄O_2_(C2)) were estimated assuming a pre-apnea mixed venous oxygen saturation and partial pressure of 75% and 40 mm Hg, respectively.

### Statistics

Unpaired two-tailed *t*-test was used to assess differences between fin types. Paired-sample two-tailed *t*-test was used to assess differences between the 50- and 100-m DYN. The agreement between *C* at 50 and at 100 m was assessed by Bland–Altman analysis. Ordinary linear regression analysis was performed between the DYN personal best distance and the reciprocal of *C* (as *d*_max_ = *E*_max_/*C*). Weighted least squares regression analysis was used to model the time course of the post-emersion excess V̇O_2_, applying a 1/*y*^2^ weighting to reduce the influence of the initial high V̇O_2_ values, which may contain greater measurement error. The difference in the prevalence of a negative Cv̄O_2_ value between C1 and C2 was assessed with the chi-square test. The level of significance was set at *P* < 0.05. The statistical package Prism 9 (GraphPad Software, La Jolla, CA) was used.

## RESULTS

**FIGURE 2:**
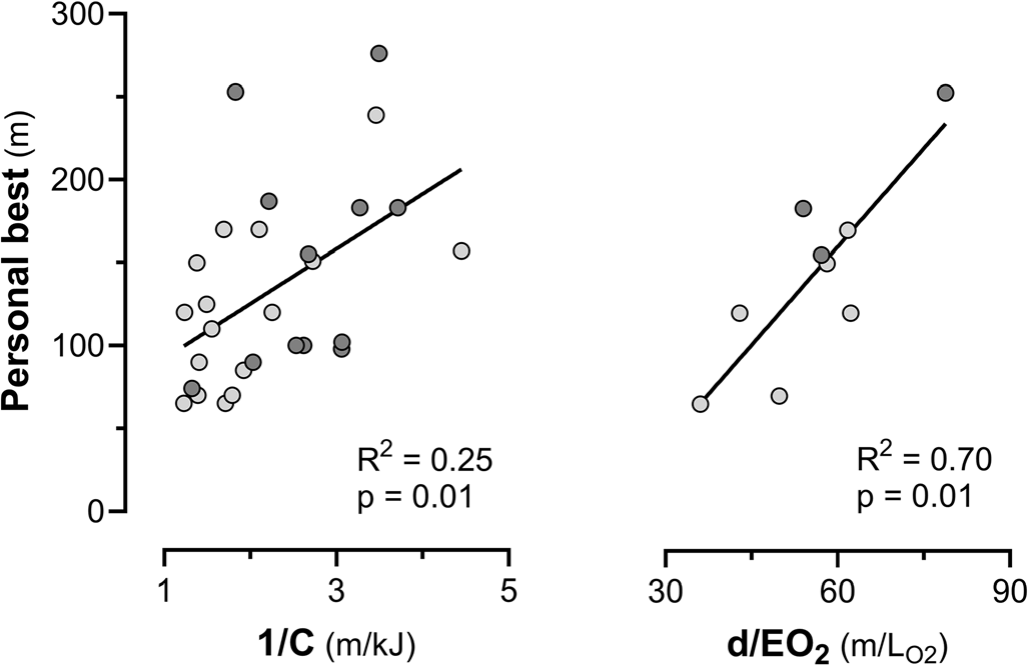
Personal best distances in dynamic apnea with fins (light gray: bi-fins; dark gray: monofin) correlated better with the meters swum per kJ expended above resting (1/*C*) (*left panel*) than with the meters swum per liter of O_2_ store depletion (*d*/EO_2_) estimated with the computational approach C1 (*right panel*), both assessed during the 50-m trial. Note the different number of data points: *n* = 28 for the left panel (10 subjects using bi-fins, 6 the monofin, and 6 both) and *n* = 9 for the right panel (6 subjects using bi-fins and 3 the monofin). Restricting the left panel to the same *n* = 9 participants would have abolished the correlation between personal best and 1/*C* (*R*^2^ = 0.11, *P* = 0.38).

Subjects had a TLC of 7.4 ± 1.1 L. All subjects completed the 50-m DYN (10 using bi-fins, 6 using the monofin, 6 using both; *n* = 28 observations). Measurements during the 50-m trials are reported in Table 1. ELa and O_2_debt were higher with bi-fins than with the monofin, thus also the derived parameters *E* and *C*. The subjects who underwent CO rebreathing (six using bi-fins and three using the monofin, *n* = 9) had an Hb_mass_ of 91 ± 129 g, a blood volume of 5.1 ± 0.9 L, and a TLC of 7.3 ± 1.0 L. Due to the small sample size, no comparison between fin types was performed in this subgroup. The personal best DYN distance was moderately related to the reciprocal of *C* at 50 m, but more strongly related to the distance covered per unit of EO_2_ (estimated with the computational approach C1) at 50 m (Fig. 2).

Seven subjects completed also the 100-m DYN (three with bi-fins, three with the monofin, and one with both; *n* = 8 observations), and their data were pairwise compared with their corresponding 50-m trial (Table 2). The speed and thus *C* and *Ė* did not change between the 50- and 100-m trials (*P* = 0.81), showing high agreement (bias, 0.00 ± 0.04 kJ·m^−1^ and 0.04 ± 0.15 L·min^−1^; 95% limits of agreement, −0.07 to +0.08 kJ·m^−1^ and −0.33 to +0.26 L·min^−1^). [La] increased by only +0.6 ± 0.4 mM from 0 to 50 m (*P* = 0.01), but notably by +1.7 ± 0.8 mM from 50 to 100 m (*P* < 0.001). Thus, ELa was four times higher in the 100-m trial than in the 50-m trial.

**TABLE 2:** Paired comparison of parameters measured at the end of 50- and 100-m dynamic apneas (*n* = 8 observations, three subjects used bi-fins, three used the monofin, and one used both).

	0 m	50 m	100 m
Time (s)	0	46.8 ± 4.5***	90.8 ± 6.0
Speed (m·s^−1^)	—	1.08 ± 0.11	1.11 ± 0.07
P_A_O_2_ (mm Hg)	125 ± 6	64 ± 7***	37 ± 6
P_A_CO_2_ (mm Hg)	27 ± 7	45 ± 7*	47 ± 7
SpO_2_	100% ± 0%	91% ± 2%***	71% ± 9%
[La] (mM)	1.1 ± 0.1	1.5 ± 0.5***	3.2 ± 1.3
ELa (L)	—	0.12 ± 0.11**	0.49 ± 0.30
O_2_debt (L)	—	1.23 ± 0.35***	2.13 ± 0.58
*E* (L)	—	1.35 ± 0.43***	2.62 ± 0.83
*Ė* (L min^−1^)	0.34 ± 0.05	1.68 ± 0.49	1.72 ± 0.56
*Ė* (mL·kg^−1^·min^−1^)	4.5 ± 0.7	23 ± 7	23 ± 8
*C* (kJ·m^−1^)	—	0.45 ± 0.16	0.44 ± 0.16
*C* (J·kg^−1^·m^−1^)	—	5.9 ± 2.2	5.9 ± 2.3
Δ*L* (L)	—	0.55 ± 0.7***	0.76 ± 0.8

The starting values at 0 m are also shown for reference.

*P < 0.05 versus 100 m.

**P < 0.01 versus 100 m.

***P < 0.001 versus 100 m.

Among those who performed the 100-m trial, *n* = 6 subjects underwent the Hb_mass_ measurement as well (three using bi-fins and three using the monofin). For these, the repartition among the various energy compartments as a function of distance is reported in Table 3, both for the C1 and the C2 computational approach (see Methods). There were no differences between C1 and C2 parameters at 50 m; however, at 100 m, C2 tended to provide lower EPCr values and to overestimate the EO_2_ and Δv̄ compared with C1 (*P* = 0.06), resulting in negative Cv̄O_2_. Interestingly, when comparing the *relative* energy contributions between 50 and 100 m, ELa/*E* doubled, EO_2_/*E*(C1) decreased, whereas EPCr/*E*(C1) remained constant and represented the second largest energy source in both distances.

**TABLE 3:** Paired comparison of the energy balance repartition among the various energy stores at the end of 50- and 100-m dynamic apneas (*n* = 6 subjects, three with bi-fins and three with the monofin).

	50 m	100 m
Δ*L* (L)	0.54 ± 0.8***	0.73 ± 0.8
Δ*m* (L)	0.05 ± 0.01***	0.10 ± 0.03
Δ*a* (L)	0.03 ± 0.01**	0.09 ± 0.03
Δv̄ (L)		
(C1)	0.20 ± 0.03***	0.38 ± 0.09
(C2)	0.21 ± 0.25**	0.79 ± 0.43
EO_2_ (L)		
(C1)	0.82 ± 0.10***	1.31 ± 0.17
(C2)	0.83 ± 0.19***	1.72 ± 0.37
EPCr (L)		
(C1)	0.52 ± 0.39**	1.00 ± 0.61
(C2)	0.51 ± 0.16*	0.59 ± 0.19
Cv̄O_2_ (mL·l^−1^)		
(C1)	98 ± 18***	51 ± 20
(C2)	92 ± 77**	−63 ± 135
Negative Cv̄O_2_		
(C1)	0/6	0/6
(C2)	0/6*	4/6****
ELa/*E*	10% ± 5%***	21% ± 5%
EO_2_/*E* (C1)	58% ± 19%*	47% ± 13%
EPCr/*E* (C1)	31% ± 20%	32% ± 15%

*P < 0.05 versus 100 m.

**P < 0.01 versus 100 m.

***P < 0.001 versus 100 m.

****P < 0.05 versus C1.

## DISCUSSION

In this study, we quantified the total energy expended during dynamic apnea with fins by assessing post-emersion excess V̇O_2_ and peak blood lactate, as well as its repartition among the three bioenergetic systems, thanks to the assessment of Hb_mass_ and TLC. To our knowledge, this is the only study evaluating the energy cost of free underwater swimming, without the additional drag and weight of SCUBA gear and tank. The high agreement in the energy cost per unit distance between 50 and 100 m suggests that this approach is internally consistent and provides a plausible estimate of total *E*.

At the speeds attained in the present study, the energy cost of DYN with bi-fins (7.4 J·m^−1^·kg^−1^) was similar to that extrapolated for surface fin swimming from the *C* versus speed relationships reported by Zamparo et al. (17) (7.8 J·m^−1^·kg^−1^), whereas it resulted slightly lower with the monofin (5.5 vs 6.8 J·m^−1^·kg^−1^). It is noteworthy that our subjects were of mixed DYN experience, whereas those in Zamparo et al. (17) were highly competitive swimmers (NCAA Division I), likely possessing a more efficient swimming technique. The subgroup capable of performing the 100-m trial, which had greater DYN experience and presumably better technique, demonstrated a slightly lower energy cost than in Zamparo et al. (17) not only with the monofin (5.4 vs 6.8 J·m^−1^·kg^−1^), but also with bi-fins (6.5 vs 7.8 J·m^−1^·kg^−1^). From these comparisons, we can conclude that, at similar swimming skill, the energy cost of DYN i) is lower with the monofin and with better swimming technique, aligning with anecdotal DYN experience and surface swimming data (1), and ii) is likely lower than in surface swimming at the same speed, consistent with hydrodynamic theory. In fact, full submersion reduces wave drag (1), and the more horizontal position provided by the neck counterweight might reduce also pressure drag (15).

In this study, we accounted for several sources of O_2_debt overestimation, such as the O_2_ consumed by the respiratory muscles during recovery, the energy expended between the end of the DYN and the start of the first inspiration, the exclusion of up to two initial outlier breaths, and the use of a weighed curve-fitting procedure. We exclude a reduction in muscular efficiency due to acidosis (42) because this effect would have increased with breath-hold duration and thus should have yielded a greater *C* at 100 m compared with 50 m. Nevertheless, *C* moderately predicted maximal DYN distance, although the EO_2_ depletion per unit distance proved to be a better predictor (Fig. 2). This suggests that oxygen-sparing mechanisms may be at least as important as an efficient swimming technique in determining maximal DYN distance.

DYN is a typical physiological unsteady state, as exercise duration is too short to allow attainment of a metabolic steady state (43), and it is performed in a closed system, with no gas exchange with the external environment. In these conditions, an oxygen deficit is necessarily incurred, which is paid back at the end of the DYN (O_2_debt in Tables 1 and 2). O_2_debt includes EPCr, that is, the alactic oxygen debt (44), and EO_2_, that is, the replenishment of the body oxygen stores (30). O_2_debt represented the major fraction of *E*, and the remainder was provided by the anaerobic lactic energy system (ELa). This latter could be determined from the increase in peak [La] above resting, as the energy equivalent of [La] accumulation is known (32), with strong experimental support in a variety of conditions such as running (45), swimming (46), during exercise transitions in normoxia (47) and in hypoxia (48), and recently also in sinusoidal cycling (49). A remarkable increase in [La] is a common observation in DYN (3–9). Overall, the contribution of [La] was small in the 50-m trial, especially with the monofin (Table 1), whereas it was by far more important in the 100-m trial (Table 2).

**FIGURE 3:**
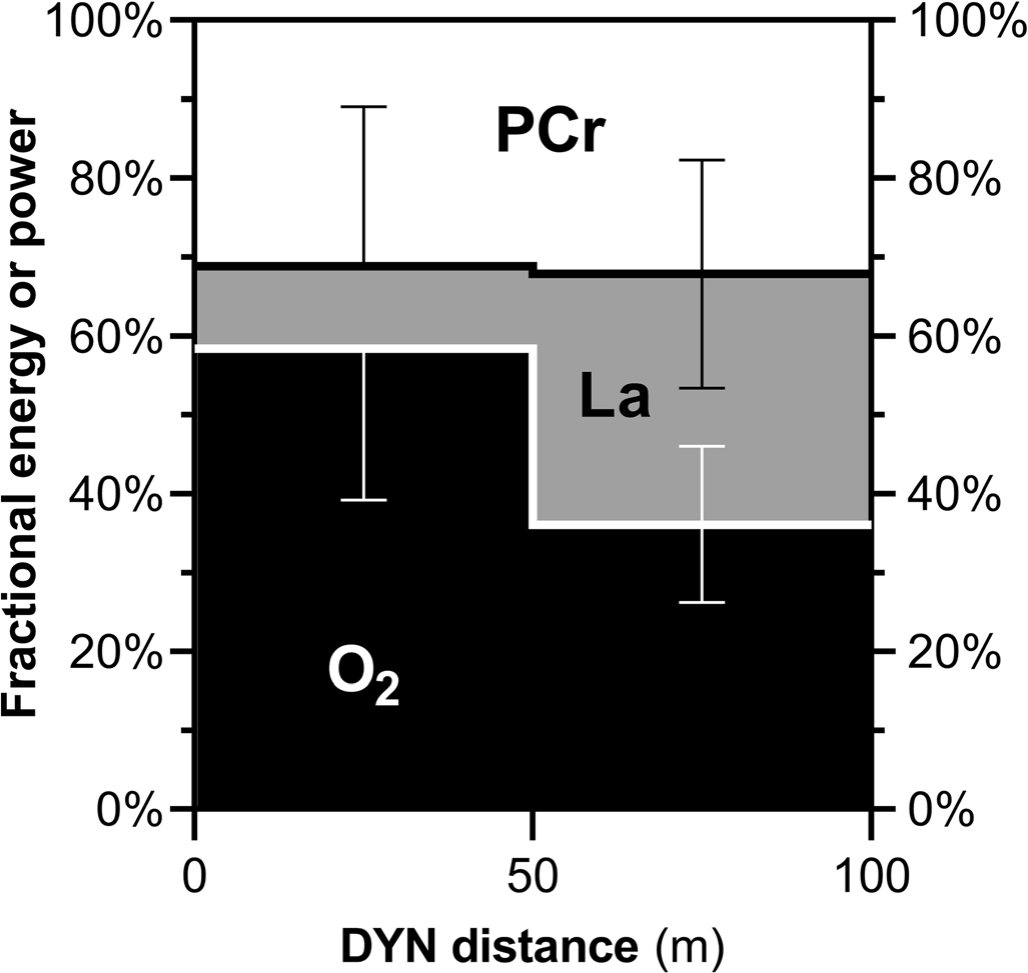
The fractional metabolic energy or power (O_2_, aerobic; La, anaerobic lactic; PCr, anaerobic alactic) utilized between 0–50 m (first histogram) and 50–100 m (second histogram) of dynamic apnea with fins (DYN). The O_2_ and PCr contributions were estimated using the computational approach C1. The second histogram was derived from the difference between the data measured at 100 and at 50 m shown in Table 2. Three subjects used bi-fins, three used monofin, and one used both (*n* = 8 observations).

In the subset of subjects who underwent Hb_mass_, we analyzed also the repartition of *E* among the various energy stores. These include EPCr, ELa, and EO_2_ (lung, blood, and myoglobin O_2_ stores). Changes in lung (Δ*L*) and arterialized (Δ*a*) oxygen stores were assessed with an established approach (30,34,38). In this study, however, it was not possible to obtain independent values of EPCr and of the changes in venous and muscle oxygen stores (Δv̄ and Δ*m*, respectively) without hazardous assumptions. In particular, for Δv̄, we used two different computational approaches, based on published kinetics of arteriovenous O_2_ difference (40) (approach C1) or of PCr hydrolysis (41) (approach C2) during a moderate exercise transition. These two approaches should provide similar values under conditions of adequate O_2_ availability and CO_2_ washout. This was the case for the 50-m trial, but not for the 100-m trial (Table 3), in which C2 tended to provide lower EPCr and higher EO_2_ and Δv̄ compared with C1, notably resulting in a negative Cv̄O_2_. This negative Cv̄O_2_ clearly indicates that approach C2 should be rejected as it underestimates EPCr contribution at 100 m, which must be higher than during eupneic exercise. Approach C1, although speculative, provided a credible Cv̄O_2_ at 100 m, compatible with Cv̄O_2_ values reported during light exercise in severe hypoxia (50,51). Collectively, these approaches suggest that fundamental bioenergetic changes occur in the second half of the 100-m trial. The energetics of the “second half” of the 100-m trial can be quantified from approach C1 as the difference between the energy contributions at 100 and 50 m (Fig. 3).

Figure 3 suggests that the relative contributions of EO_2_ and EPCr are similar to those of eupneic exercise in the first 50 m (from 0 to 47 s). However, from 50 to 100 m (from 47 to 91 s), EO_2_ fell, and this fall was almost exclusively compensated by ELa. As a result, the contribution of EPCr became higher than that of a eupneic exercise of the same duration. It is tempting to integrate this overshoot of ELa and EPCr into a diving response (6,52), where reduced blood flow to the working muscles may force them to rely more on anaerobic metabolism. ELa has traditionally been considered the main, if not the sole, anaerobic energy source in DYN, whereas the contribution of EPCr has rarely been acknowledged (15) or has been confined to short dives (22). However, a greater than expected EPCr contribution should not be surprising, as hypoxia has been shown to enhance PCr hydrolysis even during steady-state submaximal eupneic exercise (53). Moreover, the hydrolysis of PCr to creatine and inorganic phosphate buffers one H^+^ (54), making an increased reliance on EPCr appealing not only for O_2_ sparing but also for pH control. Thus, we speculate that interventions increasing the relative contribution of EPCr could enhance DYN performance. In this context, a low intramuscular *τ*, typical of endurance training, could be detrimental for DYN, as it reduces EPCr in favor of EO_2_ (equation 10). This could explain why a positive relationship between general aerobic fitness and DYN performance has never been established (15), as well as recent findings from a 5-month aerobic–anaerobic training intervention, where maximal DYN distance increased only during the anaerobic phases of the training, whereas in the aerobic phases, it either remained unchanged or even tended to decrease (55).

### Limitations

A methodological source of *C* overestimation could stem from measuring O_2_debt through the V̇O_2_ off-kinetics. Although current breath-by-breath technology has been thoroughly tested during an on-transient, less is known about its accuracy during an off-transient. Notably, longer *τ* with unchanged ΔV̇O_2_ (and thus higher area under the curve) have been reported during the off-transient compared with the on-transient in moderate exercise (56), which contrasts to bioenergetic principles (57). The Douglas bag technique offers the potential to clarify this issue in future studies. Second, the complete partitioning of energy stores by the approach C1 relied on a limited subset of subjects and multiple assumptions in the absence of Cv̄O_2_ measurement. Therefore, this latter part remains exploratory. However, it is important to note that approach C2 is independent of Cv̄O_2_ measurement, and its refutation clearly indicates that PCr utilization during DYN is higher than expected.

## CONCLUSIONS

The energy cost of dynamic apnea with fins is compatible with published values for surface swimming and hydrodynamic theory, with consistently lower values observed for the monofin compared with bi-fins. The distance swum per liter of O_2_ store depleted showed a stronger correlation with personal best DYN distance than *C*, suggesting that oxygen-sparing mechanisms are at least as critical as an efficient swimming technique. At 100 m, both the EPCr and ELa contributions were higher than those observed in analogous eupneic aerobic exercises, suggesting the presence of a diving response. Phosphocreatine metabolism may be an overlooked yet crucial component, as well as a potential target for training or nutritional interventions to enhance DYN performance. These results highlight the bioenergetic uniqueness of underwater swimming in apnea.
